# An efficient approach for limited-data chemical species tomography and its error bounds

**DOI:** 10.1098/rspa.2015.0875

**Published:** 2016-03

**Authors:** N. Polydorides, S.-A. Tsekenis, H. McCann, V.-D. A. Prat, P. Wright

**Affiliations:** 1 School of Engineering, University of Edinburgh, Edinburgh EH9 3JL, UK; 2 Instituto Nacional de Technica Aerospacial, Madrid, Spain; 3 Electrical Engineering, University of Manchester, Manchester M60 1QD, UK

**Keywords:** tomography, image reconstruction, regularization

## Abstract

We present a computationally efficient reconstruction method for the limited-data chemical species tomography problem that incorporates projection of the unknown gas concentration function onto a low-dimensional subspace, and regularization using prior information obtained from a simple flow model. In this context, the contribution of this work is on the analysis of the projection-induced data errors and the calculation of bounds for the overall image error incorporating the impact of projection and regularization errors as well as measurement noise. As an extension to this methodology, we present a variant algorithm that preserves the positivity of the concentration image.

## 1. Introduction

The concerted effort to create efficient energy technologies with reduced greenhouse gas generation, and to implement them on an industrial scale, has already resulted in the identification of numerous pollutant reduction strategies, such as biomass-derived fuels and their application in aero engines, ammonia-based energy storage, high-efficiency modes of automotive engine operation and fuel cell technology. Many more such options will be identified over the coming years. However, building innovative measurement systems to underpin the development of these technologies depends critically upon the sensitivity and resolving power of the *in situ* diagnostics. Chemical species tomography (CST) addresses this challenge by imaging quantitatively the concentration of chemical particles in gaseous media and exhaust plumes [1].

In CST, data acquisition typically entails a dense array of collimated laser beams propagating through the medium at various angles spanning the half circle. Measuring the intensity of these beams at their sources and diametrically positioned detectors yields the attenuation information within a noise margin, and that relates linearly, under some assumptions, to the chemical species concentration profile at the measurement plane. When a dense sampling of the domain is feasible, a large number of beams and many projection angles are used, and the image can then be stably reconstructed using Fourier transform-based methods like the popular filtered back-projection algorithm [2]. There are, however, harsh industrial environments where collecting many measurements is deemed impractical, allowing only a sparse beam arrangement and a small number of projection angles [3]. This is the paradigm known as limited data tomography and it is typically addressed in the context of algebraic image reconstruction and compressed sensing algorithms whenever the anticipated solution has limited support, i.e. it has a sparse profile (see e.g. [4,5]). In this work, we focus on the case where the number of projection data is substantially less than the degrees of freedom in the image we seek to reconstruct, rendering the inverse problem severely underdetermined. In addition, the sought chemical species concentration images are expected to be smooth, almost dense as a result of the gas mixing and combustion phenomena [6]. Such imaging problems are known to be ill-posed as they lack uniqueness, although a unique image can still be computed subject to enforcing some form of regularization. As discussed in some detail in [7], the choice of regularization is ultimately linked to whether the resulting inverse problem is discrete ill-posed or rank deficient. As the underlying attenuation model falls within the linear regime of the Beer–Lambert Law, the problem is well suited to the Tikhonov regularization [8], as well as iterative algorithms based on the Landweber iteration, exploiting their convergence properties in solving linear, ill-posed problems [9]. A drawback of these methods is their computational inefficiency in handling large underdetermined high-dimensional systems, as they still rely on inverting dense square matrices in high dimension.

Various experimental studies [10–14] have affirmed the sensitivity of light attenuation measurements in near and mid infrared radiation to soot and carbon dioxide particles within a jet's exhaust plume and motivate the application of tomography for *in situ* characterization. Although the gases are in motion, high-speed or simultaneous data acquisition provides static observation conditions, allowing for time-lapse imaging. Assuming negligible optical scattering, the amount of beam energy absorbed at a point is thought to be proportional to the gas density there. Effectively, the attenuation of a beam with density *p*>0 along an infinitesimal segment dℓ is [1]

1.1 dp=−pc(ℓ) dℓ,dℓ∈ℓ,

where *c* is the two-dimensional chemical species concentration function. Let **r**_*s*_ denote the start point of the ℓth beam; typically the position of the ℓth source, and **r**_*d*_ its end at the corresponding detector such that |ℓ|=|**r**_*s*_−**r**_*d*_| is the length of the beam. Further, let *p*_*s*_ to be the intensity of the beam leaving **r**_*s*_ and *p*_*d*_ the intensity of the beam arriving at **r**_*d*_, then by integrating (1.1) over the path of the beam yields

1.2 $$\int_{\ell }d\ell c(\ell )=-\int_{{\text{r}}_{s}}^{{\text{r}}_{d}}dp\frac{1}{p}=\log\left(\frac{p_{s}}{p_{d}}\right),$$

where *p*_*s*_≥*p*_*d*_≥0, and log⁡p denotes the natural logarithm of *p*. The imaging problem is then to estimate a bounded function $c:\Omega \to {ℜ}_{+}$ from a set of *m* noise contaminated data $\left\{\log(p_{s_{1}}/p_{d_{1}}),\ldots ,\log(p_{s_{m}}/p_{d_{m}})\right\}$. Before addressing this inverse problem, we make a brief remark on the impact of noise on the synthesized data compared with the actual measurements {*p*_*s*_*i*__,*p*_*d*_*i*__}. If *p*_*s*_ is known without uncertainty and *p*_*m*_ contains additive noise *n*, then $y^{*}=\log(p_{s}/p_{d})$ denotes the exact data and $y=\log(p_{s}/p_{d}+n)$ the noisy. Subtracting the one from the other yields

$$|y^{*}-y|=\left|\log\frac{p_{d}}{p_{d}+n}\right|=\left|\log\left(1+\frac{n}{p_{d}}\right)\right|\leq \left|\frac{n}{p_{d}}\right|,$$

indicating that the synthesis of the logarithmic data suppresses the levels of noise in the actual measurements. The presence of *p*_*d*_ in the denominator suggest using a high-intensity radiation, which is of course consistent with having a high signal to noise ratio in the measurements. Populating the path concentration integrals in (1.2) for many beams over the range of angles in [0,*π*) yields the linear operator equation

1.3 y=Ac+n,

where Ac is the Radon transform data of *c*, and *n* some additive noise corrupting the data. The mathematical problem of reconstructing *c* from *y* based on (1.3) is well studied and analysed in various textbooks, mostly in the context of X-ray computed tomography (e.g. [2,15–17]). Its theory postulates that a reconstruction of *c* is feasible and stable subject to the sufficiency of the data *y*. In other words, the Radon operator has a continuous inverse that leads to a unique solution provided that there are enough data to resolve the degrees of freedom in the image. In the limited-data paradigm, however, a stable inversion is no longer feasible without some form of regularization [15]. In this paper, we show that an effective re-parametrization of the unknown in conjunction with a basic regularization scheme can yield a stable, unique solution at a significantly reduced computational cost. Our approach is fundamentally based on projecting onto a low-dimensional image-feature subspace, an operation that inevitably incurs some information loss. This so-called discretization error is a data component that is typically assumed small enough to be neglected. Part of the scope of this work is to quantify its impact on the image error, particularly when the dimension of the physical domain is large and the number of data is very small by comparison. Discretizing the Beer–Lambert law on an *N*×*N* grid of square pixels, with *N*^2^≫*m* yields an underdetermined system of linear equations

1.4 y=Ac+n,

where *A*∈ℜ^*m*×*N*^2^^, with *m*=rank(*A*) and *n* is additive zero-mean Gaussian noise of magnitude *δ*=∥*n*∥, and covariance matrix *Γ*_*n*_. Without any loss of generality we assume that the system is normalized so that ∥*A*∥=1, and that *A* admits a singular value decomposition *A*=*UΣV* ′ where *U*∈ℜ^*m*×*m*^ and *V* ∈ℜ^*N*^2^×*N*^2^^ are orthogonal matrices and *Σ*∈ℜ^*m*×*N*^2^^ is a diagonal holding the singular values of *A* in non-ascending order 1≥*σ*_2_≥…≥*σ*_*m*_. In our notation prime denotes transposition. Expressing *V* and *Σ* like

$$V=[V_{m}\;|\;V_{N^{2}-m}],\;V_{m}\in {ℜ}^{N^{2}\times m},\;\Sigma =[\Sigma_{m}|0],\;\Sigma_{m}\in {ℜ}^{m\times m},$$

it is easy to see that *A* admits an expansion in a truncated basis *A*=*UΣ*_*m*_*V*_*m*_′. The columns of *U* span the range of *A*, those of *V*_*N*^2^−*m*_ its null space N(A) and $V_{m}\in N^{⊥}(A)$. Despite being underdetermined, we remark that the *m* singular values of *A* reduce at a slow rate and thus the value of *σ*_*m*_ is maintained well above zero.

An appropriate method for reconstructing a unique solution from the underdetermined model (1.4) is by formulating the Tikhonov problem, using, for example, a smoothness imposing regularization matrix *L*∈ℜ^*N*^2^×*N*^2^^ similar to that considered in [8]. This is equivalent to solving the augmented least-squares problem

1.5 $$\arg\min_{c}{∥\left(\begin{array}{c} A\\ \sqrt{\lambda }L \end{array}\right)c-\left(\begin{array}{c} y\\ 0 \end{array}\right)∥}^{2},$$

for a positive parameter λ, whose solution

1.6 $${\hat{c}}_{\lambda }={\left(A^{\prime }A+\lambda L^{\prime }L\right)}^{-1}A^{\prime }y$$

can be computed directly by inverting an *N*^2^×*N*^2^ matrix. By contrast, our methodology yields image reconstructions *without performing any computations in* *N*^2^ *dimensions*.

## 2. Model-based prior information

**Figure 1.**
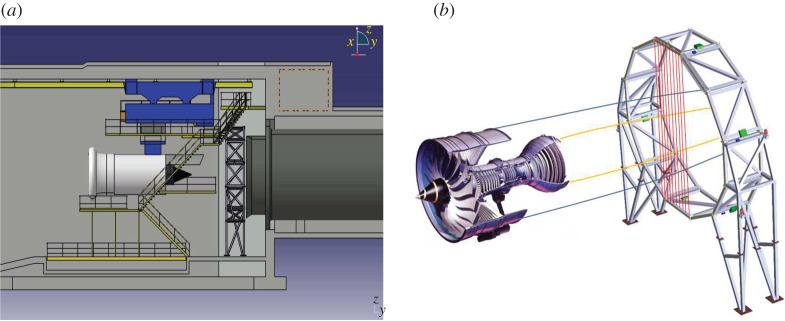
(*a*) A schematic of the FLITES experimental set-up at INTA testing facilities indicating the position of the engine, the optical ring and the detuner for gas extraction. (*b*) An oblique view of the engine and the optical plane indicating the beams from one projection angle. (Online version in colour.)

At the FLITES experiment [18], 126 light sources and detectors are mounted on a dodecagonal ring structure encompassing the imaging domain of interest *Ω*=[−0.75,0.75]×[−0.75,0.75] m, which incorporates the gas plume. The measurement plane is normal to the plume propagation axis at a distance of 2 m from the engine's nozzle as shown in figure 1. At the near field, the flow is predominantly axial with a small dispersion, while the detuner positioned immediately after the optical ring vents the engine exhaust, preventing secondary backflows in the measurement plane. In these conditions, we may assume a free turbulent jet model and simulate an ‘expected’ plume trajectory and velocity field at the measurement plane [19,11] or indeed adopt a more sophisticated fluid model if necessary [20]. In a recent study [7], the authors propose using a squared exponential prior that is consistent with turbulent flow mixing models while preserving the smoothness of the concentration profiles. The correlation between the magnitude of the velocity and the concentration of the particles in the plume hints for making a Gaussian assumption on the anticipated concentration profiles. The use of smoothness imposing prior models on this particular problem was originally suggested in [21], and here we extend it to accommodate flow-specific prior information.

**Figure 2.**
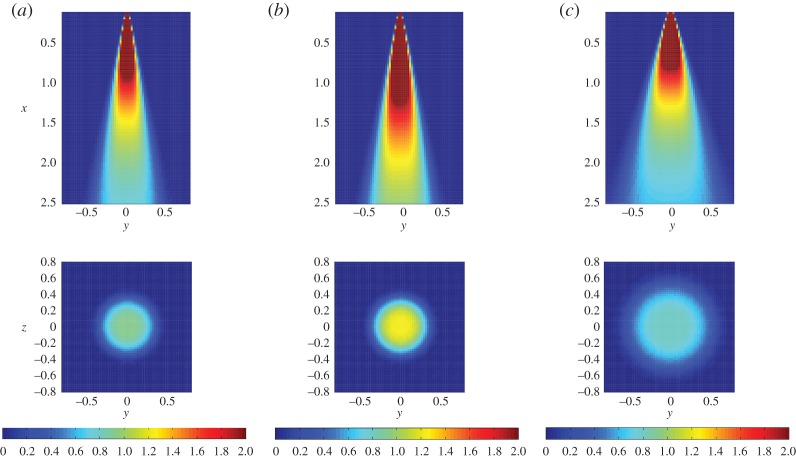
Lateral and vertical cross sections of plume concentration profiles from the super-Gaussian prior model assuming a circular nozzle of diameter *d* fixed at *x*=0.1 and the measurement plane at *x*=2 m (bottom images). For the three plumes, the respective flow parameters are *d*=0.3 m, *θ*=0.2 rad (*a*), *d*=0.4 m, *θ*=0.2 rad (*b*) and *d*= 0.4 m, *θ*=0.3 rad (*c*). In all cases, the fluid velocity at the nozzle is *u*_0_=250 m s^−1^. (Online version in colour.)

This Gaussian assumption is consistent with the experimental observations reported in [12,10], although these make reference to variant Gaussian functions modified to have a flatter top, which motivates the use of the so-called super-Gaussian functions (figure 2)

2.1 $$c_{0}(x,\varrho )=c_{\max}(x)\exp\left[-2{\left(\frac{\varrho }{x\;\tan\theta }\right)}^{\eta }\right],\;\eta >2,$$

where *x*>0 is the distance along the direction of propagation, *ϱ* is the cross-jet radial distance from the jet's centreline, *θ*>0 is the half-angle of the plume cone and $c_{\max}$ is the maximum concentration level at the centreline of the plume that is assumed to scale linearly to the plume velocity there. From the momentum conservation principle [22], if the jet nozzle has a circular shape with diameter *d* and the gas velocity there is *u*_0_ we can approximate the maximum centreline velocity as

2.2 $$u_{\max}(x)=\frac{1}{x}\frac{d}{\tan\theta }u_{0},\;x>0,$$

indicating that $u_{\max}$ decreases inversely proportional with the distance form the nozzle, while the velocity away from the centreline is

2.3 $$u(x,\varrho )=u_{\max}(x)\exp\left[-2\left(\frac{\varrho^{2}}{x^{2}\;\tan^{2}\theta }\right)\right].$$

Based on this flow model, the concentration image of a certain chemical species at the measurement plane, can be expressed as a random field from a normal probability density function

2.4 $$p_{C}(c)\propto \exp\left[-\frac{1}{2}{\left(c-c_{0}\right)}^{\prime }\Gamma_{c}^{-1}(c-c_{0})\right],$$

where *c*_0_ is the expected concentration and *Γ*_*c*_ a positive definite covariance matrix.

## 3. Subspace projection

The subspace projection relies on the hypothesis that there exists a potentially low-dimensional basis of functions that captures the dominant features of the sought image, and as such this relies explicitly on the available prior information about the solution [16,23]. Suppose c*∈ℜ^*N*^2^^ is the high-dimensional image and *Π*∈ℜ^*N*^2^×*N*^2^^ the orthogonal projection operator for functions in ℜ^*N*^2^^ onto a low-dimensional subspace

$$S≐\{\Phi r\;|\;r\in {ℜ}^{s}\},\;s\ll N^{2},$$

spanned by a small number of linearly independent basis functions {*ϕ*_1_,…,*ϕ*_*s*_} comprising the columns of the tall matrix *Φ*∈ℜ^*N*^2^×*s*^. As we discuss in the following section, there are various options for choosing these functions, and although it is not absolutely necessary, for stability reasons a choice of orthonormal basis is often preferred [16]. Here we consider the case for *Φ*′*Φ*=*I* and *ΦΦ*′=*Π*, where *Π* is idempotent. Further let r*=*Φ*′c* be the optimal coefficients in the approximation of the targeted image on *S* and $\hat{r}$ the computed solution of the projected, low-dimensional inverse problem. Our methodology consists of two steps: the projection of the high-dimensional image in the low-dimensional subspace inducing r* and the numerical solution of the projected inverse problem yielding $\hat{r}$. This process can be diagrammatically depicted as

3.1 $$c^{*}\overset{\text{approximation}}{\underset{\text{error}}{\to }}\Pi c^{*}=\Phi r^{*}\overset{\text{computational}}{\underset{\text{error}}{\to }}\Phi \hat{r},$$

indicating the two sources of errors affecting the solution. The total image error $∥c^{*}-\Phi \hat{r}∥$ comprises the subspace approximation error, depending on the skill of the basis to express c*, and the computational error which reflects the performance of the image reconstruction algorithm to approximate r* given the properties of the low-dimensional inverse problem and the impact of noise in the data. To estimate this error consider that any bounded image in ℜ^*N*^2^^ can be decomposed as

3.2 c=Πc+(I−Π)c,

where *Πc*=*Φr* for a unique *r*. Denoting by *w*_*c*_=(*I*−*Π*)*c* the subspace approximation error, otherwise put the component of *c* that does not belong in *S*, the linear model for the measurements (1.4) becomes

3.3 $$y=A(\Pi c+w_{c})+n,$$

yielding the additional data error term *Aw*_*c*_. To quantify this error further we project *w*_*c*_ onto N(A) using the orthogonal projection operator *P*=*V*_*N*^2^−*m*_*V*_*N*^2^−*m*_′, such that

3.4 $$w_{c}=Pw_{c}+(I-P)w_{c}.$$

As $Pw_{c}\in N(A)$ then the projected model for the low-dimensional variable *r*∈ℜ^*s*^ simplifies to

3.5 $$y=A\Phi r+Aq_{c}+n,$$

where *q*_*c*_=(*I*−*P*)*w*_*c*_. In regard to its magnitude, there is little to be said about the projection-induced data error *Aq*_*c*_, aside the rather obvious upper bound

3.6 $$∥Aq_{c}∥\leq ∥A∥∥q_{c}∥\leq ∥I-P∥∥w_{c}∥\leq ∥I-\Pi ∥∥c∥\leq ∥c∥,$$

as ∥*I*−*P*∥=∥*I*−*Π*∥=1. Ultimately, to estimate the overall image error we have

$$c^{*}-\Phi \hat{r}=c^{*}-\Pi c^{*}+\Pi c^{*}-\Phi \hat{r}=c^{*}-\Pi c^{*}+\Phi r^{*}-\Phi \hat{r},$$

and using the triangle inequality and the orthogonality of *Φ* we obtain

3.7 $$∥c^{*}-\Phi \hat{r}∥\leq ∥w_{c}∥+∥r^{*}-\hat{r}∥.$$

Clearly, the first component is the subspace approximation error and it relies entirely on the choice of basis *Φ*, which in turn reflects the credibility of the prior information one has about c*. To quantify the second term in (3.7), we must first specify $\hat{r}$ by formulating an appropriate inverse problem. Let *B*∈ℜ^*m*×*s*^ a low-dimensional projected model matrix with *m*≤*s*≪*N*^2^, then the model (3.5) is expressed as

3.8 $$y=Br+\varepsilon ,\;\text{where}\;\varepsilon =Aq_{c}+n,$$

with *B*=*AΦ*. As ∥*A*∥=∥*Φ*∥=1 then we can see immediately that ∥*B*∥≤1, while from [24], the *i*th singular value of *B*, denoted as ${\tilde{\sigma }}_{i}$, relates to that of the high-dimensional *A* by ${\tilde{\sigma }}_{i}\leq \sigma_{i}$ for *i*=1,…,*m* and, therefore, we can deduce that $1>{\tilde{\sigma }}_{1}\geq {\tilde{\sigma }}_{2}\geq \cdots \geq {\tilde{\sigma }}_{m}>0$. Unfortunately, this condition does not guarantee a small condition number *κ*(*B*) despite that 1/*σ*_*m*_ is small. In fact, it can be shown that for *s*<*N*^2^, *κ*(*B*)≥*κ*(*A*), which does not exclude *B* from being singular. We now have to formulate an inverse problem for the low-dimensional variable *r* based on the projected model in (3.8) and the prior information on the high-dimensional variable *c* from (2.4). The additive error *ε* inherits the Gaussian properties of the measurement noise *n* but it is shifted in mean by an unknown *Aq*_*c*_. Theoretically this term can be estimated by simulation as outlined in [17]; however, in many practical situations this tends to be neglected. The reduced model likelihood then becomes

3.9 $$p(y|r)\propto \exp\left[-\frac{1}{2}{\left(Br-y\right)}^{\prime }\Gamma_{n}^{-1}(Br-y)\right].$$

For *c*_0_∈ℜ^*N*^2^^ the mean of the flow-based prior probability density *p*_*C*_(*c*) in (2.4) then

$$\Pi c_{0}=\Pi E[c]=\Pi E[\Phi r+w_{c}]=\Phi E[r]=\Phi r_{0},$$

where E[c] denotes the expectation of *c*, and similarly

$$\Gamma_{c}=E[cc^{\prime }]=E[\Phi rr^{\prime }\Phi^{\prime }]=\Phi \Gamma_{r}\Phi^{\prime }.$$

If *Γ*_*c*_=λ^−1^*I*, for a positive λ then by the orthogonality of *Φ* we get *Γ*_*r*_=*Φ*′*Γ*_*c*_*Φ*=λ^−1^*I*, hence in forming the posterior density of $\hat{r}$ conditioned on *y* through Bayes’ rule, and tracing its unique maximum *a posteriori* estimator, yields the low-dimensional problem

3.10 $$\arg\min_{r}\{∥Br-y{∥}^{2}+\lambda ∥r-r_{0}{∥}^{2}\}$$

with solution

3.11 $${\hat{r}}_{\lambda }={\left(B^{\prime }B+\lambda I\right)}^{-1}(B^{\prime }y+\lambda r_{0}).$$

The computational error $∥r^{*}-\hat{r}∥$ depends on how accurately the solution of the reduced inverse problem (3.11) approximates r* given the necessity of regularization and the various noise components embedded in *ε*. Assuming the general case where ${\tilde{\sigma }}_{m}\approx 0$ and λ>0, we can investigate how the estimator ${\hat{r}}_{\lambda }$ is aided by the prior knowledge of *r*_0_ and how it is corrupted by the measurement noise and approximation errors in *ε*. Let *y*=y*+*ε* and suppose the prior guess on the solution satisfies *r*_0_=r*+*δr*, for an arbitrary *δr*, then by (3.11) we obtain

$$\begin{array}{rl} {\hat{r}}_{\lambda } & ={\left(B^{\prime }B+\lambda I\right)}^{-1}(B^{\prime }y+\lambda r_{0})\\ & ={\left(B^{\prime }B+\lambda I\right)}^{-1}[B^{\prime }(Br^{*}+\varepsilon )+\lambda (r^{*}+\delta r)]\\ & ={\left(B^{\prime }B+\lambda I\right)}^{-1}[(B^{\prime }B+\lambda I)r^{*}+B^{\prime }\varepsilon +\lambda \delta r]\\ & =r^{*}+{\left(B^{\prime }B+\lambda I\right)}^{-1}(B^{\prime }\varepsilon +\lambda \delta r). \end{array}$$

Rearranging, and taking norms we obtain the computational error upper bound for (3.7)

$$\begin{array}{rl} ∥{\hat{r}}_{\lambda }-r^{*}∥ & =∥{\left(B^{\prime }B+\lambda I\right)}^{-1}(B^{\prime }\varepsilon +\lambda \delta r)∥\\ & \leq ∥{\left(B^{\prime }B+\lambda I\right)}^{-1}B^{\prime }∥∥\varepsilon ∥+\lambda ∥{\left(B^{\prime }B+\lambda I\right)}^{-1}∥∥\delta r∥\\ & =\max_{i=1,\ldots ,m}\left\{\frac{{\tilde{\sigma }}_{i}}{{\tilde{\sigma }}_{i}^{2}+\lambda }\right\}(∥q_{c}∥+∥n∥)+\frac{\lambda }{{\tilde{\sigma }}_{m}^{2}+\lambda }∥\delta r∥, \end{array}$$

as $\lambda \gg {\tilde{\sigma }}_{m}\approx 0$ and therefore

3.12 $$∥r^{*}-{\hat{r}}_{\lambda }∥\leq \max_{i=1,\ldots ,m}\left\{\frac{{\tilde{\sigma }}_{i}}{{\tilde{\sigma }}_{i}^{2}+\lambda }\right\}(∥q_{c}∥+∥n∥)+∥r^{*}-r_{0}∥.$$

Introducing to the general error bound (3.7), we obtain

3.13 $$∥c^{*}-\Phi {\hat{r}}_{\lambda }∥\leq ∥w_{c}∥+\max_{i=1,\ldots ,m}\left\{\frac{{\tilde{\sigma }}_{i}}{{\tilde{\sigma }}_{i}^{2}+\lambda }\right\}(∥q_{c}∥+∥n∥)+∥r^{*}-r_{0}∥,$$

which indicates that the overall image error in our approach is the sum of the approximation error norm and the norm of the discrepancy between the prior-based expectation and the true image. While the error terms ∥*w*_*c*_∥ and ∥r*−*r*_0_∥ depend exclusively on the available *a priori* information and the sufficiency of the reduced basis, the error amplification term $\max_{i=1,\ldots ,m}\{{\tilde{\sigma }}_{i}/\left({\tilde{\sigma }}_{i}^{2}+\lambda \right)\}>1$ can be precomputed for any choice of λ. Given the slow reduction rate in the large singular values of *B* the amplification may turn out to be small. Note, however, that a small computational error $∥r^{*}-{\hat{r}}_{\lambda }∥$ does not imply a small overall error $∥c^{*}-\Phi {\hat{r}}_{\lambda }∥$, as the next section demonstrates.

## 4. Choice of approximation basis and a special case

**Figure 3.**
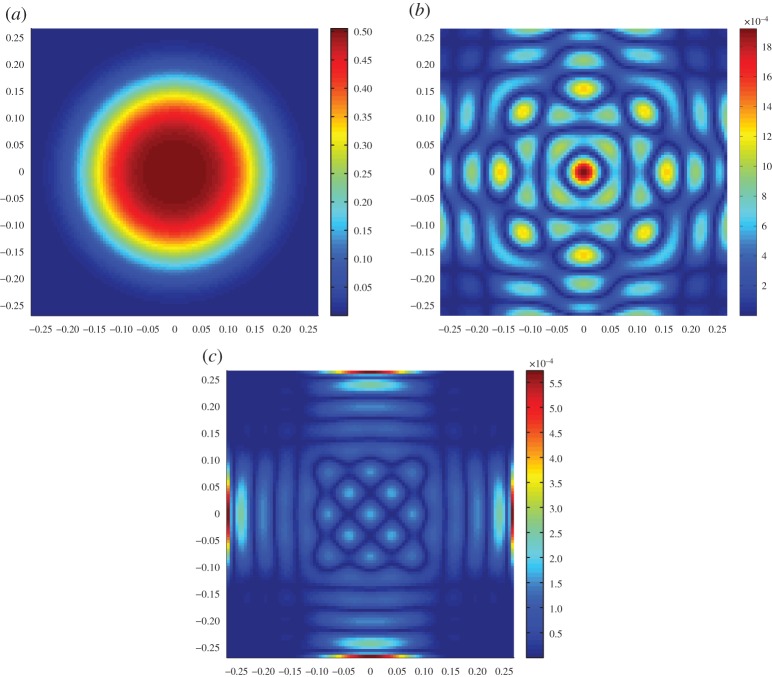
(*a*) A high-dimensional image c* and next its projection errors *w*_*c*_ in *s*=64 (*b*) and *s*=144 (*c*) discrete cosine transform functions. The relative errors ∥c*−Πc*∥/∥c*∥ are 2×10^−3^ and 3×10^−4^, respectively. (Online version in colour.)

We have seen that projecting the unknown high-dimensional image into a low-dimensional subspace reduces the computational complexity by replacing the large matrix *A* with a smaller matrix *B*. This computational advantage causes an increase in the ‘noise’ of the data from *n* to *ε*, in manifestation of the subspace approximation error *w*_*c*_. Moreover, this projection may lead to a rank deficient inverse problem and a new matrix with dispersed singular values, despite the clustering in those of the original large matrix. Choosing the basis *Φ* appropriately is thus instrumental in the image reconstruction process, in the sense that it controls the approximation error *w*_*c*_, but it also has an impact on the computational error. One conclusion that becomes apparent even as early as this stage of the investigation is that the conventional local basis functions with pixel-wise constant support, often adopted by default, might not always be the best basis to model the unknown image when having limited measurements. We propose the use of global basis functions, a smooth basis of orthonormal functions that satisfy the prior (2.4) [16]. Subject to a sufficiently large *s*, this basis can accommodate a large range of functions, whose features include the expected concentration (see e.g. figure 3). We note that the computational advantage of this approach compared with solving the smoothness imposing high-dimensional Tikhonov regularization problem (1.5) may come at a cost of a higher projection approximation error, as the admissible smoothness of the image is explicitly enforced in terms of this basis. Moreover, other non-smoothness related priors in the context of Bayesian inference, or affine constraints in optimization schemes, may prove challenging to cast in the form of conforming global bases, and in those circumstances it might be easier to revert to the conventional pixel-based basis.

Let us now look closer into computing the minimum norm solution for the high-dimensional model (1.4) formulated as

$$\hat{c}=\arg\min_{c\in {ℜ}^{N^{2}}}∥c∥\;\mathrm{such}\;\mathrm{that}\;y=Ac+n.$$

Our intent is to show that this is a special case of the subspace projection method with *Φ*=*V*_*m*_ (*s*=*m*) in which case $\kappa (B)=\kappa (A)=\sigma_{m}^{-1}$, *Π*=*P*^⊥^, and thus the basis is optimal in terms of the computational error. Effectively,

$$Aq_{c}=A(I-P)w_{c}=A\Pi w_{c}=0,$$

and hence the measurement error due to the subspace approximation error vanishes leaving *ε*=*n*. This unique estimator can be computed rather efficiently, and stably, by inverting only an *m*×*m* square matrix *B*=*AV*_*m*_. Given that *AA*′ is invertible by virtue of *κ*(*A*) being small, then $\hat{c}=A^{\prime }{\left(AA^{\prime }\right)}^{-1}y$ can alternatively be computed by solving the low-dimensional least-squares problem

$$\hat{r}=\arg\min_{r\in {ℜ}^{m}}∥AV_{m}r-y{∥}^{2}.$$

If *Σ*_*m*_∈ℜ^*m*×*m*^ is the non-zero block of *Σ* that holds the singular values of *A*, then it is straightforward to show that $\hat{r}=\Sigma_{m}^{-1}U^{\prime }y$ and therefore $\hat{c}=V_{m}\hat{r}$. Note that in this case the computational error attains its minimum possible value $∥r^{*}-\hat{r}∥=\tau \delta$. To see this recall that r*=*V*_*m*_′c* and

$$\begin{array}{rl} \hat{r} & =\Sigma_{m}^{-1}U^{\prime }y=\Sigma_{m}^{-1}U^{\prime }(AV_{m}r^{*}+A(I_{N^{2}}-P)w_{c}+\varepsilon )\\ & =\Sigma_{m}^{-1}U^{\prime }(U\Sigma_{m}V_{m}^{\prime }V_{m}r^{*}+U\Sigma_{m}V_{m}^{\prime }(I-P)w_{c}+\varepsilon )\\ & =r^{*}+V_{m}^{\prime }(I-P)w_{c}+\Sigma_{m}^{-1}U^{\prime }\varepsilon \\ & =r^{*}+\kappa (A)n, \end{array}$$

hence combining with the approximation error we have

$$∥c^{*}-\Phi \hat{r}∥\leq ∥c^{*}-\Pi c^{*}∥+∥V_{m}(\hat{r}-r^{*})∥\leq ∥c^{*}-\Pi c^{*}∥+\kappa (A)\delta .$$

**Figure 4.**
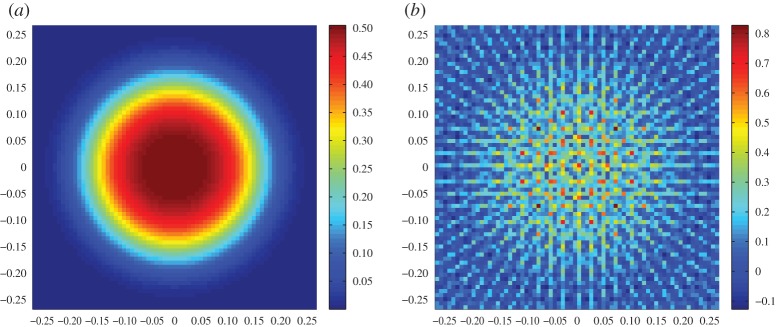
(*a*) A target image c* consistent with the prior flow-concentration model (2.4) and (*b*) its projection *V*_*m*_*V*_*m*_′c*. The relative approximation error ∥c*−Πc*∥/∥c*∥ is 0.66 with *s*=*m*=126 functions and the condition number of the square matrix *B*=*AV*_*m*_ is *κ*(*B*)=2.5. (Online version in colour.)

Choosing the basis *Φ*=*V*_*m*_ is thus optimal for minimizing the computational error, as $∥\hat{r}-r^{*}∥\to 0$ as δ→0, and no regularization error occurs. Unfortunately, this advantage is diminished by the fact that the basis *V*_*m*_ is unsuitable for reconstructing the anticipated smooth solutions, see for example, the projection of an image into this basis in figure 4, and results in a very large approximation error ∥c*−Πc*∥.

## 5. A variant algorithm to impose positivity

Having reduced the dimension of the inverse problem through the projection to a low-dimensional basis, we proceed to suggest a modification that precludes the solution from attaining non-positive values. This is aimed at preserving the feasibility of the images, as chemical concentration is by definition non-negative. In particular, the attenuation model is reformulated in terms of the logarithm of the unknown concentration function [25]. In this context, denoting z=log⁡c then the original model (1.4) becomes nonlinear in the new parameters

$$y=Ae^{z}+n,$$

which can then be linearized around a point *z*_*i*_∈*S* to

$$y=Ac_{i}+AC_{i}(z-z_{i})+n,$$

where *C*_*i*_=*diag*(*c*_*i*_) and *c*_*i*_=*e*^*z*_*i*_^. Denoting *K*(*z*_*i*_)=*AC*_*i*_ and *y*(*z*_*i*_)=*y*−*Ac*_*i*_+*K*(*z*_*i*_)*z*_*i*_, we cast the *i*th linear model for *y* at the iterate *z*_*i*_ as

5.1 $$y(z_{i})=K(z_{i})z+n.$$

Note that the new unknown variable is still in the high-dimension, so similar to the unconstrained case we can project it onto *S* to get *z*=*Πz*+*w*_*z*_ where now *Πz*=*Φr*, and *B*(*z*_*i*_)=*K*(*z*_*i*_)*Φ*, yielding the low-dimensional model

5.2 $$y(z_{i})=B(z_{i})r+\varepsilon ,\;\varepsilon =K(z_{i})w_{z}+n.$$

Assuming once again that the basis functions *Φ* spanning *S* are orthonormal then a positive subspace projected estimator of the image ${\hat{c}}^{+}$ can be obtained iteratively using the following algorithm:

(i) Initialize *z*_*i*_∈*S* and compute *c*_*i*_=*e*^*z*_*i*_^,
(ii) for *i*=1,2,3,…
  (a) Compute matrix *B*(*z*_*i*_)=*K*(*z*_*i*_)*Φ* and vector *y*(*z*_*i*_),
  (b) Compute ${\hat{r}}_{i+1}^{+}$ using (3.11) with *B*=*B*(*z*_*i*_) and *y*=*y*(*z*_*i*_),
  (c) Update $z_{i+1}=\Phi {\hat{r}}_{i+1}^{+}$,
(iii) end
(iv) ${\hat{c}}^{+}=e^{z_{i}}$.

Although we do not derive error bounds for this positively constrained estimator, we note that as *c* can no longer admit zero values, some information loss is incurred in the transformation from *c* to *z*, and, therefore, the bounds of the previous section no longer apply. In this case, only the positive part of the function *c* can be uniquely mapped to a corresponding function z=log⁡c, while a zero concentration value will typically be assigned a very small yet still positive value.

## 6. Numerical results

In order to evaluate the performance of the proposed algorithms and verify the image error bound (3.13), two sets of image reconstruction experiments were performed using simulated data infused with white Gaussian noise of standard deviation equal to 5% of the mean measurement value. In both instances, we consider concentration functions that are similar but not equal to the prior guess *c*_0_. This prior is based on model (2.1) with *x*=2 m, *c*_*max*_=5 and *θ*=0.2 rad, and it appears in figure 3 plotted in a domain *Ω*=[−0.26,0.26]×[−0.26,0.26] with normalized distance units. The first three target images are of the form

**Figure 5.**
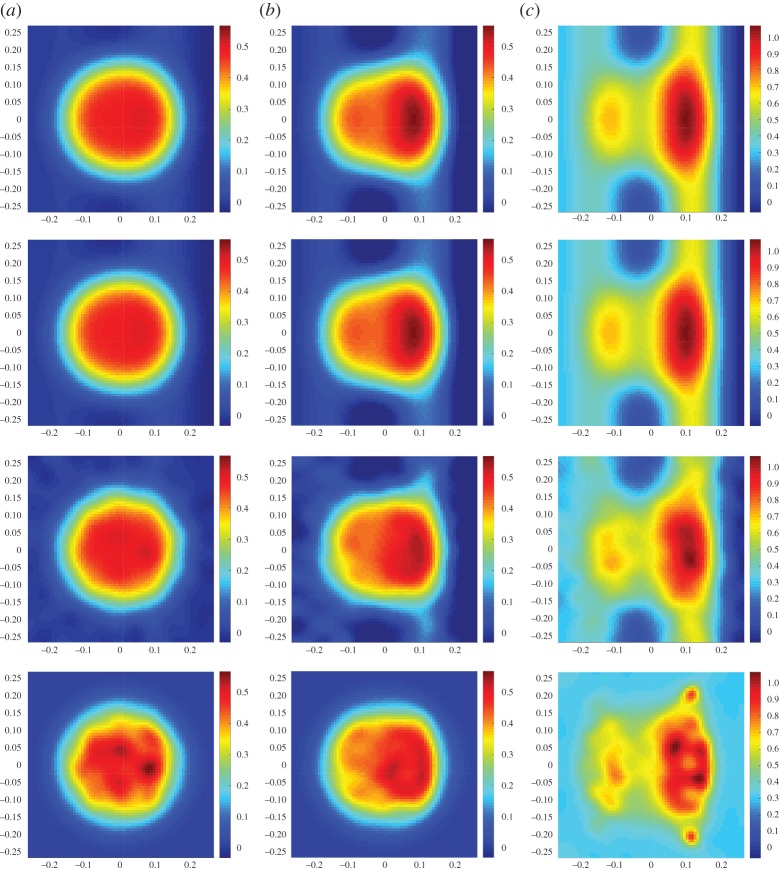
(*a*–*c*) are, respectively, the reconstruction results for *c*_1_, *c*_2_ and *c*_3_ concentration functions from equation (6.1). At the top row, the true concentration targets at fine discretization with *N*=100, and below their projection *Πc*_*i*_, projected image reconstruction $\Phi {\hat{r}}_{i}$ and fourth iteration of the positively constrained estimator ${\hat{c}}_{i}^{+}$ all in resolution *N*=70, assuming a subspace of *s*=144 discrete cosine transform functions. (Online version in colour.)

**Figure 6.**
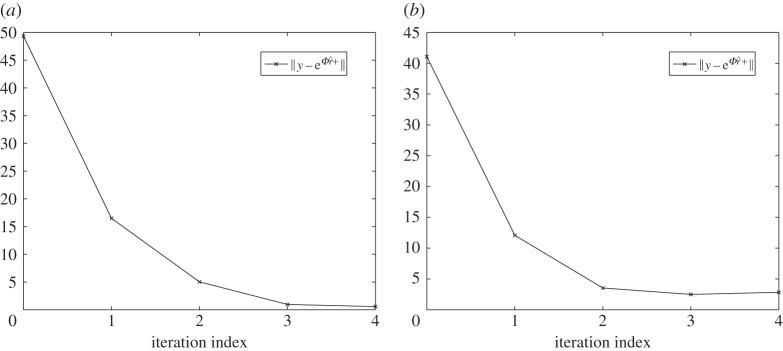
Convergence of the positively constrained algorithm on the reconstruction of ${\hat{c}}_{1}^{+}$ in (6.1) (*a*) and ${\hat{c}}_{5}^{+}$ in (6.2) (*b*).

6.1 $$c_{i}(\varrho_{1},\varrho_{2})=c_{0}+\beta_{i}(\sin(3\phi_{3}(\varrho_{1},\varrho_{2}))-\sin(3\phi_{4}(\varrho_{1},\varrho_{2}))),\;i=1,2,3,$$

where *ϕ*_*j*_ denotes the *j*th function in the approximation basis (e.g. the *j*th column of *Φ*), *β*_1_=0.25, *β*_2_=1 and *β*_3_=3 is a parameter that controls the deviation of the target function from *c*_0_, and *ϱ*_1_,*ϱ*_2_ are the coordinates on the plane of measurement. The definitions *c*_1_, *c*_2_ and *c*_3_ were chosen to yield a small approximation error, e.g. ∥*w*_*c*_∥/∥*c*∥≈10^−4^, and consequently the approximation error component in the data *Aq*_*c*_ is small enough to be ignored, ∥*Aq*_*c*_∥/∥y*∥≈10^−4^. The measurements have been computed on a fine 100×100 square grid model of *Ω*, while for the imaging problem a coarser 70×70 grid was used. Consistent with the FLITES experiment, 126 attenuation measurements were computed from six projection angles, and a model matrix *A*∈ℜ^126×4900^ was assembled. On the coarse grid, we also define *Φ*∈ℜ^4900×144^ whose columns represent an orthonormal basis of *s*=144 discrete cosine basis functions. Forming the low-dimensional projected model yields a rank-deficient matrix *B*∈ℜ^126×144^ with *κ*(*B*)≈10^16^. For each of the targeted concentration functions *c*_1_, *c*_2_ and *c*_3_, we have computed low dimensional estimators ${\hat{r}}_{1}$, ${\hat{r}}_{2}$ and ${\hat{r}}_{3}$ using (3.11). The results obtained are summarized in table 1 and the respective images appear in figure 5. A quick glance at the table confirms the derived error bounds $∥c-\Phi \hat{r}∥\leq ∥w_{c}∥+∥r^{*}-\hat{r}∥$ and $∥r^{*}-\hat{r}∥\leq ∥r^{*}-r_{0}∥$ in all three cases, where r*=*Φ*^†^*c* is the optimum low-dimensional solution, and *r*_0_=*Φ*^†^*c*_0_ the projection of the prior concentration on *S*. The value of the regularization factor λ used in each case was estimated heuristically as outlined at the end of this section. As anticipated, when the approximation error is very small, the overall reconstruction error scales to the discrepancy between the true image and the prior guess ∥r*−*r*_0_∥, and, therefore, a lower λ value is more appropriate when this discrepancy can be large. Note, however, that the overall image error $∥c-\Phi \hat{r}∥$ is not proportional to ∥r*−*r*_0_∥, despite that ∥*w*_*c*_∥≈0.005 remains fixed in all tests. For comparison, a positively constrained solution ${\hat{c}}^{+}$ is also obtained by performing four iterations of the algorithm in section 5. The algorithm exhibits a fast convergence (figure 6), but the value of the error $∥{\hat{c}}^{+}-c∥$ appears to be significantly higher compared with that of the unconstrained solution.

**Table 1.** Three simulated experiments on target concentrations with negligibly small approximation error. Each row corresponds to a different *c*_*i*_ from (6.1) and the subscript on *c* is omitted for clarity in the presentation. In all cases, ∥*w*_*c*_∥≈0.005 and ∥*Aq*_*c*_∥≈0.001. The image error $∥c-\Phi \hat{r}∥$ increases with ∥r*−*r*_0_∥, while the value of λ reduces.

*i*	∥*c*∥	∥r*−*r*_0_∥	λ	$∥c-\Phi \hat{r}∥$	$∥r^{*}-\hat{r}∥$	$∥c-{\hat{c}}^{+}∥$
1	15.856	01.060	0.134	0.783	0.783	1.359
2	16.369	04.240	0.016	1.035	1.035	3.566
3	20.272	12.722	0.006	1.659	1.659	9.061

To investigate the impact of a significant approximation error on the image reconstruction, another set of simulated experiments were performed using three different target concentrations. Assuming the same prior for consistency, these new target concentration functions are

**Figure 7.**
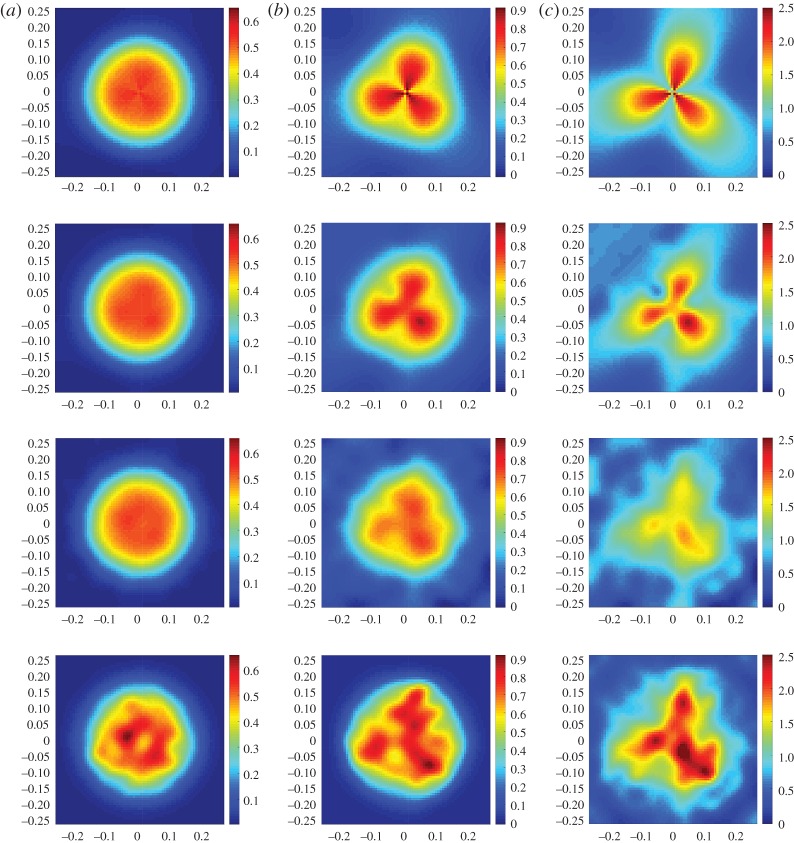
(*a*–*c*) are, respectively, the reconstruction results for *c*_4_, *c*_5_ and *c*_6_ concentration functions from equation (6.2). At the top row, the true concentration targets at fine discretization with *N*=100, and below their projection *Πc*_*i*_, projected image reconstruction $\Phi {\hat{r}}_{i}$ and fourth iteration of the positively constrained estimator ${\hat{c}}_{i}^{+}$ all in resolution *N*=70, assuming a subspace of *s*=144 discrete cosine transform functions. (Online version in colour.)

6.2 $$c_{i}(\xi ,\vartheta )=c_{0}+\beta_{i}(e^{-\cos(3\vartheta )}+e^{-\sin(3\vartheta )})e^{-\xi },\;i=4,5,6,$$

where (*ξ*,*ϑ*) are the polar coordinates of (*ϱ*_1_,*ϱ*_2_). The coefficients *β*_4_=0.01, *β*_5_=0.1 and *β*_6_=0.5 control the size of the prior guess discrepancy ∥r*−*r*_0_∥. Contrary to the previous tests, these incur a significant approximation error in the adopted basis, as it can be seen by comparing the images at first and second rows of figure 7. In this case, we consider a gradually increasing approximation error in conjunction with an increasing prior discrepancy. The results of these experiments are tabulated in table 2, but the reconstructions of *c*_4_, *c*_5_ and *c*_6_ show that while λ should be reduced in increasing ∥r*−*r*_0_∥ in order to relax the influence of the prior guess, the increasing approximation error *Aq*_*c*_ in the data reinstates a high regularization parameter value. This being the primary difference in the two tests, the error bounds $∥c-\Phi \hat{r}∥\leq ∥w_{c}∥+∥r^{*}-r_{0}∥$ and $∥c-\Phi \hat{r}∥\leq ∥w_{c}∥+∥r^{*}-\hat{r}∥$ were found to hold in this ∥*w*_*c*_∥≫0 case. Moreover, the errors in the constrained and unconstrained solutions are at equivalent levels, as opposed to those for ∥*w*_*c*_∥≈0, where the unconstrained estimators were profoundly superior. However, a closer look at the images in figure 7 reveals that quantitatively, the high concentration levels are more accurately reconstructed in the constrained solutions ${\hat{c}}_{5}^{+}$ and ${\hat{c}}_{6}^{+}$ despite the higher overall error.

**Table 2.** Three simulated experiments on target concentrations with significant approximation error. Each row corresponds to a different *c*_*i*_ from (6.2). Notice that the overall error $∥c-\Phi \hat{r}∥$ is less than the sum of the approximation and computational errors.

*i*	∥*c*∥	∥*w*_*c*_∥	∥*Aq*_*c*_∥	∥r*−*r*_0_∥	λ	$∥c-\Phi \hat{r}∥$	$∥r^{*}-\hat{r}∥$	$∥c-{\hat{c}}^{+}∥$
4	16.633	10.325	0.115	11.050	0.308	10.712	10.633	11.129
5	24.940	13.256	1.149	10.504	0.016	14.086	12.468	16.768
6	67.285	16.283	5.749	52.522	0.064	19.351	10.456	22.574

### (a) Choosing a value for the regularization parameter

**Figure 8.**
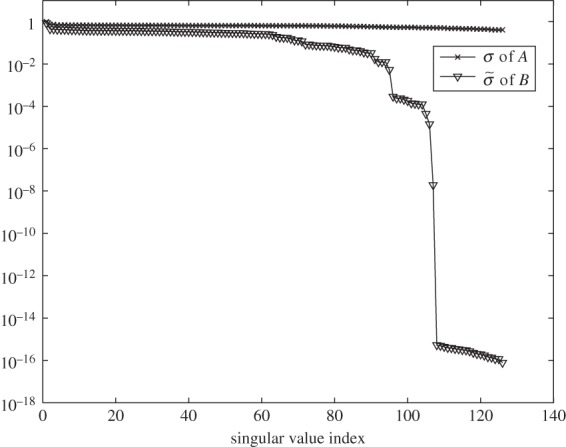
The singular values of matrices *A*∈ℜ^*m*×*N*^2^^ and *B*=*AΦ* with *Φ*∈ℜ^*N*^2^×*s*^, where *m*=126, *s*=144 and *N*=70. Note the slow rate in the reduction of the singular values of *A* and the effective rank deficiency of *B*. *Φ* consists of a basis of discrete cosine functions.

The choice of the regularization parameter λ has a significant impact on the reconstructed Tikhonov estimator ${\hat{r}}_{\lambda }$. In abstract form, this problem has been studied extensively in the context of Bayesian estimation for linear inverse problems and various approaches have been proposed on optimizing this parameter in conjunction to the noise levels in the data and the confidence one has on the prior information [16]. The generalized cross validation (GCV) and L-curve methods are among the most popular tools for choosing λ, for example Ma & Cai [26] on their implementation in chemical species tomography. These schemes refer predominantly to linear Gaussian models assuming some knowledge on the noise's first and second statistical moments. Alternatively, one may use criteria based on the singular value decomposition in the cases where the model matrices are rank deficient [27]. As in our setting, regularization is applied to the projected problem, which includes an unknown data error component *Aq*_*c*_, L-curve and GCV are not straightforward to apply. Instead, we resort in a heuristic criterion for choosing a sub-optimal λ as follows. We generate a dense grid of *M* logarithmically *equally spaced* values in the interval $\left[{\tilde{\sigma }}_{\text{rank}(B)},{\tilde{\sigma }}_{1}\right]$, where ${\tilde{\sigma }}_{\text{rank}(B)}$ is the smallest, non-zero singular value of *B*, which can be easily identified from the ‘jump’ in the singular values as illustrated in figure 8. Assigning in turn, each value in that interval to λ and solving the problem (3.11) yields a set of solutions $\left\{{\hat{r}}_{\lambda_{1}},{\hat{r}}_{\lambda_{2}},\ldots ,{\hat{r}}_{\lambda_{M}}\right\}$, from which we compute a residual vector $\Delta {\hat{r}}_{\lambda_{i}}={\hat{r}}_{\lambda_{i}}-{\hat{r}}_{\lambda_{i-1}}$, for *i*=2,…,*M* and then plot the graph of $∥\Delta {\hat{r}}_{\lambda_{i}}∥$ on a *linear scale*, as a function of λ evaluated at the midpoint of the interval [λ_*i*_,λ_*i*−1_].

**Figure 9.**
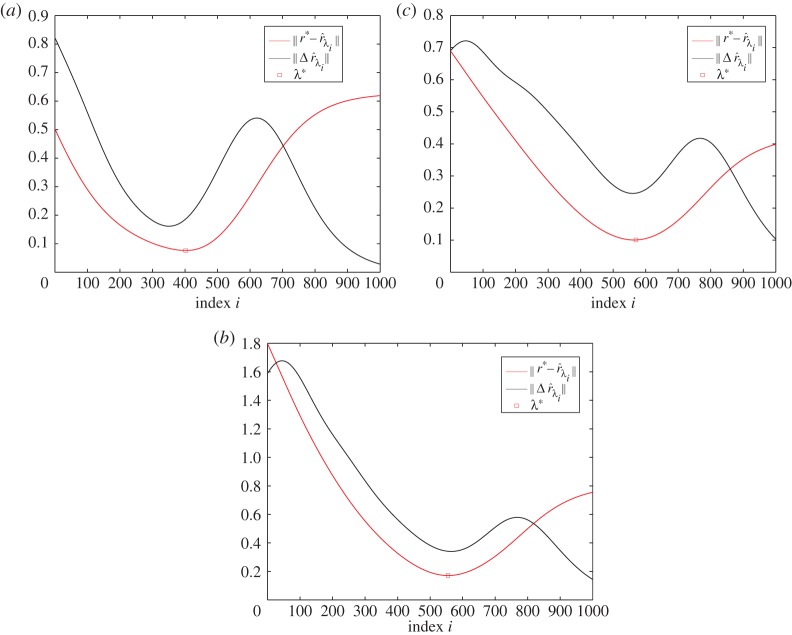
Plots of the $∥\Delta {\hat{r}}_{\lambda_{i}}∥$ and $∥r^{*}-{\hat{r}}_{\lambda_{i}}∥$ for 1000 values of λ equally spaced within [10^−4^,1]. Note the proximity of the optimal regularization parameter λ* to the argument that minimizes $∥\Delta {\hat{r}}_{\lambda_{i}}∥$. The graphs refer to the experiments in reconstructing ${\hat{r}}_{3}$ (*a*), ${\hat{r}}_{5}$ (*b*) and ${\hat{r}}_{6}$ (*c*). (Online version in colour.)

Our criterion for choosing λ from this curve is that in the neighbourhood of the optimal λ the value of $∥\Delta {\hat{r}}_{\lambda }∥$ will be minimized indicating a region of stability, in the sense that a small perturbation in λ will only cause a small perturbation on the solution ${\hat{r}}_{\lambda }$. More precisely, as $\lambda :{\tilde{\sigma }}_{\text{rank}(B)}\to {\tilde{\sigma }}_{1}$, we anticipate the residual norm to gradually reduce as regularization sets in to alleviate the instabilities caused by the rank deficiency of matrix *B*. This reduction will lead to a minimum near the optimal λ*, before the residual norm increases again in response to the regularization beginning to affect some of the larger singular values that are clustered closely together. Thereafter, we expect the $∥\Delta {\hat{r}}_{\lambda_{i}}∥$ curve to converge to zero as λ approaches ${\tilde{\sigma }}_{1}$ due to over-regularization, which prevents the solutions to deviate from the prior *r*_0_. The graphs of $∥\Delta {\hat{r}}_{\lambda_{i}}∥$ for the selection of λ in the reconstruction of *c*_3_, *c*_5_ and *c*_6_ are shown in figure 9. To demonstrate the validity of this approach we have also computed $∥r^{*}-{\hat{r}}_{\lambda_{i}}∥$ using r*=*Φ*^†^*c* for each value of λ in the same interval, regarding as optimal parameter choice λ* the argument that minimizes this error discrepancy. In reality of course $∥r^{*}-{\hat{r}}_{\lambda_{i}}∥$ is not known, but the proximity of λ* to the minimum of $∥\Delta {\hat{r}}_{\lambda_{i}}∥$ can be used as a guide for selecting a near-optimal regularization parameter.

## 7. Conclusion

This work proposes a computationally efficient solution of the inverse problem in limited-data chemical species attenuation tomography with flow model-based prior information. We have showed that projection of the unknown function in a smooth low-dimensional basis reduces drastically the dimensionality of the problem and probably yields rank-deficient linear systems that are suitable to Tikhonov regularization. This image reconstruction approach is computationally efficient as it avoids inverting high-dimensional matrices; however, it incurs errors due to the subspace projection and the need for regularization in obtaining a stable solution of the projected inverse problem. To quantify these errors and their impact on the reconstructed image, we provide an upper bound on the overall image error which incorporates the subspace approximation and regularization errors and can be used in the interpretation of the reconstructed images. In this bound the approximation error appears both as an offset as well as a component of the data error, however, the amplification factor of these errors in the image reconstruction is maintained at small levels. This framework is complemented by an variant algorithm for solving the positively constrained imaging problem and a heuristic method for selecting the required regularization parameter. The numerical simulations affirm the performance of the algorithms proposed and the validate the error bounds.
